# Effect of calcium oxide and soybean hull addition to feedlot diets containing dried distillers grains and corn stover on steer performance, carcass characteristics, and digestibility

**DOI:** 10.1093/tas/txaa105

**Published:** 2020-06-30

**Authors:** Nicholas A Lancaster, Chris R Muegge, Jose R R Carvalho, Rodrigo C Lopes, Rafael S Narumiya, Fabio Pinese, Aubrey Nickie Baird, Jon P Schoonmaker

**Affiliations:** 1 Department of Animal Sciences, Purdue University, West Lafayette, IN; 2 Department of Animal Science, Universidade Federal de Lavras, Lavras, MG, Brazil; 3 Department of Animal Science, University of Sao Paulo, Pirassununga, SP, Brazil; 4 Department of Veterinary Clinical Sciences, Purdue University, West Lafayette, IN

**Keywords:** beef feedlot, calcium oxide, digestibility, kinetics, ruminal pH, soybean hulls

## Abstract

The objective of this study was to determine the effect of soybean hulls (SBH) and/or calcium oxide (CaO) on rumen pH, digestibility, and performance of steers fed diets containing dried distillers grains with solubles (DDGS). In experiment 1, Angus × Simmental steers (*n* = 112, body weight [BW] = 364 ± 7.8 kg) were allotted to 1 of 4 diets arranged as a 2 × 2 factorial and placed in 16 pens (7 steers/pen, 4 pens/treatment, and 28 steers/treatment). Factors were SBH (0% or 30% of diet dry matter [DM]) and CaO (0% or 1%) inclusion. Basal diets contained 20% corn stover, 30% DDGS, and 4% supplement. Diets with SBH contained 14.1% or 15.0% corn and diets without SBH contained 43.9% or 44.8% corn. In experiment two, four steers (BW = 510 ± 9.8 kg) were allotted to a 4 × 4 Latin square (21 d periods) to determine the effects of CaO and SBH on ruminal pH, volatile fatty acid (VFA), nutrient digestibility, and digestion kinetics. Statistical analyses were conducted using the MIXED procedure of SAS. In experiment 1, BW did not differ among treatments (*P* ≥ 0.46). Overall carcass-adjusted gain did not differ due to SBH or CaO inclusion (*P* ≥ 0.13); however, there was an interaction (*P* = 0.01) where CaO improved gain in steers fed no SBH, but not in steers fed SBH. Steers fed SBH consumed more DM than steers not fed SBH (*P* = 0.02) and an interaction tended to occur (*P* = 0.06) where CaO increased dry matter intake in steers fed no SBH, but not in steers fed SBH. Calcium oxide increased hot carcass weight and yield grade (interaction; *P* ≤ 0.04) and tended to increase fat thickness (interaction; *P* = 0.08) in steers fed no SBH, but not in steers fed SBH. Dressing percentage, longissimus muscle area, % kidney, pelvic, heart fat, and marbling score did not differ among treatments (*P* ≥ 0.14). Total VFA concentrations were greater with SBH inclusion and with CaO addition (*P* < 0.01). Digestibility of DM, neutral detergent fiber (NDF), and acid detergent fiber (ADF) was greater with CaO addition (*P* ≤ 0.04) and NDF and ADF digestibility were greater with SBH inclusion (*P* < 0.001). Inclusion of SBH did not affect (*P* ≥ 0.26) rate of digestion (*k*_d_) or passage (*k*_p_). Addition of CaO tended to increase mean retention time (*P* = 0.09). An interaction between SBH inclusion and CaO addition occurred for *k*_d_ (*P* = 0.01), where CaO increased *k*_d_ in steers fed SBH, but decreased *k*_d_ when steers were fed no SBH. Total N excretion tended to be lower with SBH inclusion and CaO addition (*P* = 0.07). In conclusion, CaO enhances performance of cattle fed corn, DDGS, and corn stover diets, but not when corn is partially replaced by a fiber-based energy feed.

## INTRODUCTION

Inclusion of starch-based grains in high-fiber diets can result in development of negative associative effects, leading to reduced intake and poor fiber digestion (Pordomingo et al., 1991; Fieser and Vanzant, 2004). Mertens and Loften (1980) reported that fiber digestion was delayed by the addition of starch to a pH controlled in vitro incubation. This negative effect on fiber digestion was attributed to ruminal bacteria capable of digesting both starch and fiber preferentially digesting starch (Mertens and Loften 1980) and producing end products that decrease cellulase and hemicellulase activity (van der Linden et al., 1984). Distillers grains with solubles (DGS) is a highly digestible fiber energy feed. When DGS replaces a portion of corn in diets that contain 10% to 20% roughage there could be a decrease in negative associative effects on fiber digestion. However, inclusion of DGS in feedlot rations can increase dietary acidity because sulfuric acid is added in the ethanol fermentation practice (McAloon et al., 2000). Thus, cattle consuming rations containing elevated amounts of DGS can exhibit a ruminal pH as low as 5 (Felix and Loerch, 2011). This lower ruminal pH inhibits the growth, reproduction, and efficiency of fiber fermenting cellulolytic bacteria due to a decrease in their ability to attach to feed and a decrease in their ability to produce cellulases (Hoover, 1986).

Soybean hulls (SBH) are 74% neutral detergent fiber (NDF) (NRC, 1996); however, the energy value of SBH in rations can vary depending on what other components are included in the diet. Ludden et al. (1995) demonstrated that SBH fed in a starch-based feedlot ration provided only 74% the energy of corn. However, others have observed that SBH fed in high-fiber diets provides energy comparable to that of corn (Anderson et al., 1988; Garcés-Yépez et al., 1997). Additionally, Bittner et al. (2016) demonstrated that when SBH are fed in combination with DGS, SBH can provide from 99% to 106% of the energy content of corn as a result of a positive associative effect on ruminal fermentation. Digestibility of low-quality forages has increased with dietary SBH inclusion (Highfall et al., 1987; Galloway et al., 1993; Fieser and Vanzant, 2004; Orr et al., 2008). Additionally, alkali treatments to low-quality roughages to enhance digestibility has been known for years (Klopfenstein, 1978). Recent research has demonstrated that the addition of alkalizing agents such as calcium oxide (CaO) or calcium hydroxide (Ca(OH)_2_) were effective at increasing ruminal pH and enhancing fiber digestibility in feedlot steers fed 60% dried DGS diets (Nuñez et al., 2014; Lancaster et al., 2019). Consequently, we hypothesized that adding CaO to 30% dried DGS diets would increase ruminal pH and enhance a potential positive associative effect between stover, SBH, and DGS resulting in improved fiber digestibility and growth performance of the cattle.

## MATERIALS AND METHODS

Research protocols involving the utilization of animals followed guidelines set forth in the Guide for the Care and Use of Agricultural Animals in Agricultural Research and Teaching (FASS, 2010) and were approved by the Purdue Animal Care and Use Committee (protocol # 131100098 and 1304000851).

### Experiment 1

#### Animals, housing, and diets

One hundred and twelve Angus × Simmental steers (average initial body weight [BW] = 364.1 ± 7.87 kg) were used at the Animal Sciences Research and Education Center (ASREC) of Purdue University to determine the effect of SBH inclusion and CaO addition to feedlot diets containing dried DGS and stover on steer performance and carcass characteristics. Steers were weighed prior to feeding and allotted, by BW, frame size, and breed composition to one of four treatments, arranged as a 2 × 2 factorial. Steers were vaccinated against bovine rhinotracheitis, bovine viral diarrhea, parainfluenza-3, and bovine respiratory syncytial virus (Bovi-Shield GOLD FP 5; Zoetis Animal Health), against Haemophilus somnus, Pasturella, and Clostridia (Vision-7 Somnus; Merck Animal Health), and treated with an anthelmintic (Valbazen; Zoetis Animal Health) for internal and external parasites at weaning and upon feedlot entry. Steers were implanted with Revalor-XS (4 mg estradiol and 20 mg trenbolone acetate; provided courtesy of Merck Animal Health, Summit, NJ) at feedlot entry. Steers were housed in 16 pens (7 steers/pen, 4 pens/treatment, and 28 steers/treatment) located in a curtain-sided, slatted-floor finishing barn. Pen dimensions were 6.1 × 3.4 m and provided 0.9 m of bunk space per animal.

Treatments consisted of a base ration containing 30% dried DGS, 20% corn stover, and 4% vitamin/mineral supplement with corn, SBH, urea, and calcium source making up the difference. Treatments were: 1) no SBH with 0% CaO inclusion (43.9% corn, 1.9% limestone, and 0.2% urea), 2) no SBH with 1% CaO inclusion (44.8% corn, 1% CaO, and 0.2% urea), 3) 30% SBH with 0% CaO (14.1% corn and 1.9% limestone), and 4) 30% SBH with 1% CaO (15% corn). Diets are provided in Table 1. Diets were formulated to meet or exceed NRC (1996) requirements for protein, vitamins, and minerals of growing beef cattle. Corn stover was harvested at approximately 80% dry matter (DM). Corn stover was chopped in a windrow and then harvested using a silage chopper (Jaguar model 930, Claas, Columbus, IN) equipped with a flail header and stored in an air-tight silage bag (Up North, Cottage Grove, MN) until the initiation of the experiment. Calcium oxide was provided courtesy of Mississippi Lime (St. Louis, MO). Diets were delivered once daily at 0800 h and steers were allowed ad libitum access to feed and water. Feed deliveries were recorded and adjusted daily using the South Dakota State University 4-point bunk scoring system (Pritchard, 1993) to minimize the accumulation of unconsumed feed. Feed samples were taken every other week and dried in a forced air oven at 60 °C for 48 h. Dried feed samples were ground using a standard Wiley laboratory mill (1-mm screen; Arthur H. Thomas, Philadelphia, PA), and composited at the end of the experiment for analysis of crude protein (CP) (Micro-Kjeldahl N × 6.25; method 960.52; AOAC, 2006), ether extract (method 920.39; AOAC, 2006), and minerals (Ca, P, Mg, K, and S; method 968.08; AOAC, 2006). Neutral detergent fiber was determined using an ANKOM Fiber Analyzer (ANKOM Technology Corp., Fairport, NY) based on the procedure of AOAC (2006, method 2002.04) using heat-stable α-amylase (Termamyl 120 L, Type L, Novozymes A/S) and sodium sulfite. Acid detergent fiber (ADF) was determined based on AOAC (2006, method 973.18) with modifications to each procedure for use in an ANKOM Fiber Analyzer. As-fed formulations were adjusted for DM content accordingly every other week.

**Table 1.** Composition of diets (DM basis)

	No SBH		SBH	
	No CaO	CaO	No CaO	CaO
Corn	43.9	44.8	14.1	15.0
Soybean hulls	—	—	30.0	30.0
DDGS	30.0	30.0	30.0	30.0
Stover	20.0	20.0	20.0	20.0
Urea	0.2	0.2	—	—
Supplement^1^	4.0	4.0	4.0	4.0
Limestone	1.9	—	1.9	—
CaO	—	1.0	—	1.0
Nutrient composition				
Crude protein	17.4	17.4	17.2	17.3
NE_m_, Mcal/kg	2.07	2.09	1.85	1.87
NE_g_, Mcal/kg	1.22	1.23	1.04	1.06
Calcium	1.07	1.06	1.06	1.05
Phosphorus	0.52	0.52	0.47	0.47
Sulfur	0.29	0.29	0.29	0.29

NE_g_ = Net energy for gain;

NE_m_ = Net energy for maintenance.

^1^Vitamin/mineral premix contained (DM basis) 1.00% Ca, 0.45% Mg, 2.03% K, 0.25% S, 2.60 mg/kg Co, 252.56 mg/kg Cu, 2.63 mg/kg I, 96.78 mg/kg Fe, 572 mg/kg Mn, 6.32 mg/kg Se, 875.43 mg/kg Zn, 62.9 IU/g vitamin A, 7.48 IU/g vitamin D, 224 IU/kg vitamin E, 640.8 mg/kg monensin (Rumensin 80, Elanco Animal Health, Indianapolis, IN), and 198.4 mg/kg tylosin (Tylan 40, Elanco Animal Health, Indianapolis, IN).

#### Growth performance and carcass characteristics measurements

Steers were weighed on two consecutive days at the start and end of the study for the determination of initial and final BWs. During the experiment, steers were weighted monthly to monitor average daily gain (ADG). Body weights were taken prior to feeding. Scales (Tru-Test XR3000; Mineral Wells, TX) weighed to the nearest 0.9 kg (for BW <453.6 kg) or 2.3 kg (for BW >453.6 kg) and were checked for accuracy at each weigh date. Average daily gain was determined by the difference between final and initial BW divided by the number of days on feed. Dry matter intake (DMI) was recorded and used to calculate feed efficiency (gain:feed) by dividing ADG by DMI. Feedlot performance of the steers was calculated for d 0 to 86 (period 1), d 86 to slaughter (period 2), and d 0 to slaughter (overall). A common dressing percentage of 62.7% was used to calculate a final carcass-adjusted BW, and a carcass-adjusted ADG and gain:feed for period 2 and overall were also calculated. Animals were slaughtered at a commercial packing facility (Tyson Foods, Joslin, IL) when they achieved a target BW of approximately 622 kg. Hot carcass weight and dressing percentage were determined after slaughter and prior to chilling. After carcasses were chilled for 24 h, the following measurements were obtained by qualified University personnel: subcutaneous fat thickness taken between the 12th and 13th ribs, longissimus muscle (LM) area obtained by direct grid reading of the LM between the 12th and 13th ribs, internal fat (kidney, pelvic, and heart fat; %KPH) as a percentage of hot carcass weight, marbling score, and United States Department of Agriculture quality and yield grades (USDA, 1997).

#### Statistical analysis

Data were analyzed as a completely randomized design utilizing the mixed procedure of SAS (Version 9.3, SAS Inst. Inc., Cary, NC) with pen considered the experimental unit. Random effects of pen and the fixed effect of CaO addition, SBH inclusion, day, as well as the CaO addition × day, SBH × day, CaO addition × SBH inclusion, and CaO addition × SBH inclusion × day interactions were included in the model. Performance was analyzed as repeated measures by comparing four covariance structures for each variable (compound symmetric, autoregressive order one, heterogeneous autoregressive order one, and unstructured). The covariance structure that yielded the lowest Bayesian information criterion was utilized for presented results. Carcass characteristics and overall performance were analyzed using MIXED procedure of SAS as a randomized design and the model included the random effects of pen, and the fixed effect of treatment. Treatment comparisons were made using Fisher’s protected least significant difference, and the least square means statement was used to calculate adjusted means. The SLICE function of SAS was used to determine simple effects within time for repeated measures. Simple effects within day are presented in the results section. Quality grade percentages were analyzed as binary data using the GLIMMIX procedures of SAS. Statistical significance was determined when *P* ≤ 0.05 and tendencies were discussed when 0.05 < *P* ≤ 0.10.

### Experiment 2

#### Animals, housing, and diets

Four ruminally cannulated Angus × Simmental steers (average initial BW = 510.2 ± 9.79 kg) were used at the Purdue University ASREC to determine the effects of CaO addition and SBH inclusion on ruminal pH, volatile fatty acid (VFA) production, apparent digestibility, and nitrogen balance. Steers were fistulated approximately 9 mo prior to the start of the study. Steers were previously adapted to a 30% dried distillers grains with solubles (DDGS) ration and then allotted to a 4 × 4 Latin square design balanced for carry over effects, such that each experimental diet followed a different diet for each respective treatment. The four diets were identical to those in experiment 1 (Table 1) and treatments were arranged as a 2 × 2 factorial. The experimental periods were 21 d and were composed of a 16 d adaptation to the respective diet and 5 d of sampling. Steers were housed individually in 3.0 × 9.1 m outdoor pens for the first 14 d of each period. Pens were inside a three-sided barn with concrete floors covered with wood chips. On d 15, steers were moved into a climate controlled room with temperatures maintained between 17 and 21 °C and continuous lighting. Steers were individually housed in 1.0 × 2.0 m tie stalls equipped with rubber mats. The individual tie stalls were designed for total urine and fecal collections. Each respective ration was mixed individually and delivered once daily at 0800 h and steers were allowed ad libitum access to feed and water. Feed delivery, bunk management, diet sampling, and health protocol were the same as those used in experiment 1.

#### Sampling

On day 17 of each period, 100 g of Cr-mordanted stover and a pulse dose of 200 mL of a solution containing 10 g of Co-Ethylenediaminetetraacetic acid both prepared according to Udén et al. (1980), were administered into the rumen through the cannula before the morning feeding. Fecal grab samples were collected from the rectum at h 0, 12, 18, 24, 36, 48, 60, 72, 84, and 96 after dosing. Feces were weighed, homogenized and dried in a forced air oven at 60 °C for 48 h and milled through a 1-mm screen. Dried fecal grab samples were stored at room temperature for later analysis of Cr content. Rumen contents were collected via the rumen cannula at 1.5, 3, 6, 9, 12, 18, 24, 36, 48, 72, 84, and 96 h after dosing. Rumen fluid was strained through two layers of cheese cloth and pH was immediately measured and recorded using a pH meter (VWR sympHony SB70P benchtop pH meter with glass combination pH electrode, VWR International, LLC, Batavia, IL). Rumen fluid was then placed in a 50 mL conical tube, immediately capped to prevent volatilization and placed in −20 °C freezer and stored for later analysis of VFA and Co concentrations.

On day 17 of periods 3 and 4, nylon bags (40-µm pore size) containing 10 g of DM of each individual dietary feed ingredient (except supplement) were placed in the rumen to permit 0, 3, 6, 9, 12, 18, 24, 48, 72, and 96 h of incubation. Feeds were ground in a Wiley mill through a 2-mm screen and bags were placed in the rumen in reverse order such that the greatest incubation time (96 h) was placed first and the shortest incubation time (3 h) was placed last; all bags were removed at h 96. After removal, bags were immediately rinsed six times in cold water and dried at 60 °C for 48 h. Weight of the residue was determined after drying. The 0 h incubation samples were washed in the same way, but were not incubated in the rumen.

Digestibility measurements were accomplished by collecting total daily fecal output in metal pans built into the floor at the tail end of the tie stall and determined every 24 h for d 17 to 21. A subsample of total feces was collected each day and stored for later analysis of DM, CP, NDF, and ADF as described previously. Total urinary output was collected every 24 h for d 17 to 21 into metal pans built into the floor in the middle of the tie stall. The metal collection pans were sloped to one side where there was an output hole, allowing urine to flow into a plastic collection container below. One hundred mL of a 3 N HCl solution was added to the plastic collection bins prior to urinary collection to acidify the urine and avoid N volatilization and a subsample of the total urine was stored in a 50 mL conical tube at −20 °C until analysis of N.

#### Chemical analysis

For VFA analysis, ruminal fluid samples were thawed, centrifuged at 12,000 × *g* for 20 min, filtered through a 0.45 µm filter, then shell-frozen and freeze-dried after addition of 8% sodium hydroxide. Volatile fatty acids were extracted from the freeze-dried ruminal fluid, derivatized as butyl esters, and quantified on a gas chromatograph equipped with a flame ionization detector (Model 7890A; Agilent Technologies, Santa Clara, CA) according to Salanitro and Muirhead (1975). For Co and Cr analysis, approximately 250 mg of freeze-dried rumen fluid and dried feces were solubilized in a solution containing 5 mL of nitric acid, heated at 100 °C, then diluted in distilled water. Rumen fluid was analyzed for Co and feces and mordanted stover were analyzed for Cr using inductively coupled plasma mass spectroscopy (ICP-MS; Optima 5300-DV, Perkin-Elmer, Waltham, MA). Feces were analyzed for DM, CP, NDF, and ADF as described previously in experiment 1 and for organic matter (OM) which was calculated as the difference between DM and ash content (AOAC, 2006). Urine samples were analyzed for total N (block digestion followed by steam distillation), and ammonia N (steam distillation) using a Kjeltec 2300 micro-Kjeldahl Analyzer Unit (Foss Tecator, AB, Foss North America). Urinary urea output was calculated as the difference between total N output and ammonia N output. Prior to feeding and acidification of urine, fresh urine was collected and used to determine urine pH using the previously described pH meter.

#### Calculations

Digestibility of the diets was determined using the equation:

$$\begin{array}{l} \left[\frac{nutrient\;\;\;in\;\;\;feed-nutrient\;\;\;in\;\;\;feces}{nutrient\;\;\;in\;\;\;feed\;}\right]\times 100 \end{array}$$

Solid passage rate was determined by plotting concentrations of Cr in feces against the time of collection after dosing the marker and fitting the generated fecal marker excretion curve using the procedure proposed by Grovum and Williams (1973a, 1973b) based on the model of Blaxter et al. (1956). This model has two pools within the gastrointestinal tract with longer and shorter retention times, respectively, and a tubular compartment corresponding to the omasum, small intestine, and distal part of the large intestine. The model is defined as follows:

$$\begin{array}{l} if\;t>TT,\;then\;Y=Ae^{-k_{1}(t-TT)}Ae^{-k_{2}(t-TT)}, \end{array}$$

else *Y* = 0, where *Y* is Cr concentration in feces (mg/kg of fecal DM); *A* is a scale parameter; the irrational constant *e* is the base of the natural logarithm; *k*_1_ (h^−1^) is the fractional outflow rate from the reticulorumen (the smaller rate constant for the pool with a longer retention time); *k*_2_ (h^−1^) is the fractional outflow rate from the hindgut (the larger rate constant for the pool with a shorter retention time); and TT (h) represents the transit time through the omasum, small intestine, and distal part of the large intestine and can be considered the minimum time taken by a particle to transit between the point of its introduction and sampling. The time between marker dosage and feces collection is represented by *t* (h). Transit time and mean retention time of solids were estimated according to Grovum and Phillips (1973). Liquid passage rate (*k*_L_, h^−1^) was calculated by fitting a linear regression to the natural logarithm of Co concentration in the rumen liquid (mg/L) against sampling time (h), according to the method of Warner and Stacy (1968).

Ruminal DM degradation data of diets were calculated using individual in situ residue weights from individual feed ingredients. Degradation data of diets were fitted to the modified first-order kinetics equation with lag time to determine rate and extent of feed degradation (Ørskov and McDonald, 1979; Robinson et al., 1986):

$$\begin{array}{l} R(t)=U+D^{\left(-k_{d}\times (t-T0)\right)} \end{array}$$

where *R*(*t*) is the residue of the incubated material after *t* h of rumen incubation (g), *U* is the undegradable fraction (%), *D* is the potentially degradable fraction (%), *T*0 is the lag time (h), and *k*_d_ is the degradation rate (%/h). Effective degradability (g/kg) of DM was determined using the nonlinear (NLIN) parameters (*U*, *D*, and *k*_d_) calculated by the above equation and also the following equation:

$$\begin{array}{l} ED=S+D\times \left[\frac{k_{d}}{k_{p}+k_{d}}\right] \end{array}$$

where *S* is the soluble fraction (%) as determined by the samples incubated for 0 h and *k*_p_ is the rate of passage fixed at 3.0% according to Ørskov and McDonald (1979). A nonlinear regression method of SAS (SAS Inst. Inc., Cary, NC) was used to estimate degradability coefficients.

#### Statistical analysis

Data were analyzed as a 4 × 4 Latin square design utilizing the MIXED procedure of SAS (Version 9.3, SAS Inst. Inc., Cary, NC) with steer within period considered the experimental unit. Repeated measures were used to analyze rumen pH and VFA concentrations and the model included the random effects of steer and period and fixed effects of the CaO addition, SBH inclusion, time, the interaction between time and CaO addition, the interaction between time and SBH inclusion, and the interaction between time, SBH inclusion, and CaO addition. For digestibility, rate and extent of digestion, and passage rates, the model included the random effects of steer and fixed effects of CaO addition, SBH inclusion, and the interactions between period and CaO addition, between period and SBH inclusion, and between period, CaO addition, and SBH inclusion. Four covariance structures were tested for each variable (compound symmetric, autoregressive order one, heterogeneous autoregressive order one, and unstructured) and the covariance structure that yielded the lowest Bayesian information criterion was used for the presented results. Treatment comparisons were made using Fisher’s protected least significant difference, and the least square means statement was used to calculate adjusted means. The SLICE function of SAS was used to determine simple effects within time for repeated measures. Differences were considered statistically significant when *P* ≤ 0.05 and tendencies were discussed when 0.05 < *P* ≤ 0.10.

## RESULTS

### Experiment 1

Feedlot performance data are presented in Table 2. Steer BW did not differ among treatments at any point in the study and did not differ when adjusted to a common dressing percentage (*P* ≥ 0.36). Average daily gain from d 0 to 86 was greater for steers fed SBH than for steers not fed SBH (*P* = 0.03). Addition of CaO did not affect ADG from d 0 to 86 (*P* = 0.22), but an interaction occurred (*P* = 0.01) where CaO only improved ADG for steers fed no SBH. When not adjusted for carcass weights, ADG for steers during the second half of the study (d 86 to 155) and overall did not differ because of fiber inclusion or CaO addition (*P* ≥ 0.20). However, there was a tendency for an interaction for second half (*P* = 0.09) and there was and interaction (*P* = 0.01) for overall carcass-adjusted ADG. For steers fed no SBH, addition of CaO increased carcass-adjusted second half and overall ADG whereas for steers fed SBH, addition of CaO decreased carcass-adjusted second half and overall ADG. Days on feed did not differ for steers among treatments (*P* ≥ 0.39). Inclusion of SBH did not impact DMI during the first half (*P* = 0.12), but increased second half (*P* = 0.03) and overall (*P* = 0.02) DMI. Addition of CaO had no impact on DMI during any period or overall (*P* ≥ 0.19). However, an interaction between CaO addition and SBH inclusion occurred for DMI in the first half of the study (*P* = 0.03) and tended to occur during the second half of the study (*P* = 0.10) and overall (*P* = 0.06) where addition of CaO in diets with no SBH increased DMI and addition of CaO to diets with SBH decreased DMI. Gain:feed was not impacted by SBH addition during the first half of the study (*P* = 0.45), overall (*P* = 0.82), or overall carcass-adjusted (*P* = 0.69). However, from d 86 to 155 gain:feed was greatest for steers fed SBH free diets (*P* = 0.04) and carcass-adjusted second half gain:feed tended to be greatest (*P* = 0.06) for steers fed SBH free diets. Addition of CaO did not affect gain:feed at any point in the study (*P* ≥ 0.12). An interaction was noted for second half (*P* = 0.05) and carcass-adjusted second half (*P* = 0.02) gain:feed where addition of CaO to diets with no SBH increased gain:feed, while addition of CaO to diets with SBH decreased gain:feed.

**Table 2.** Effect of SBH inclusion or CaO addition on steer performance

	Treatments							
	No SBH		SBH			*P*-value		
	No CaO	CaO	No CaO	CaO	SE	Fiber^2^	Alkali^3^	F × A^4^
Weight, kg								
Day 0	362	361	363	360	7.8	0.99	0.81	0.99
Day 86	499	513	518	510	8.2	0.36	0.70	0.46
Slaughter (day 155)	612	621	623	614	13.9	0.90	0.97	0.93
Slaughter (day 155), adjusted^1^	608	628	623	612	18.7	0.99	0.80	0.87
ADG, kg/d								
Day 0 to 86	1.62^a^	1.80^b^	1.85^b^	1.77^b^	0.041	0.03	0.22	0.01
Day 86 to slaughter	1.49	1.67	1.63	1.58	0.117	0.87	0.60	0.75
Day 86 to slaughter, adjusted^1^	1.37^a^	1.74^b^	1.56^ab^	1.50^a^	0.093	0.80	0.21	0.09
Day 0 to slaughter	1.56	1.71	1.75	1.67	0.059	0.25	0.47	0.20
Day 0 to slaughter, adjusted^1^	1.66^a^	1.73^a^	1.76^a^	1.52^b^	0.045	0.22	0.13	0.01
DMI, kg/d								
Day 0 to 86	10.0^a^	10.4^a^	11.0^b^	10.2^a^	0.22	0.12	0.36	0.03
Day 86 to slaughter	11.0^a^	11.7^ab^	12.1^b^	12.6^b^	0.42	0.03	0.19	0.10
Day 0 to slaughter	10.5^a^	10.9^ab^	11.5^b^	11.2^b^	0.22	0.02	0.73	0.06
Gain:feed, kg/kg								
Day 0 to 86	0.162	0.172	0.167	0.174	0.0054	0.45	0.12	0.37
Day 86 to slaughter	0.135^ab^	0.145^b^	0.135^ab^	0.123^a^	0.0054	0.04	0.82	0.05
Day 86 to slaughter, adjusted^1^	0.125^a^	0.148^b^	0.130^a^	0.118^a^	0.006	0.06	0.43	0.02
Day 0 to slaughter	0.150	0.155	0.152	0.150	0.0054	0.82	0.82	0.90
Day 0 to slaughter, adjusted^1^	0.145	0.160	0.153	0.148	0.006	0.69	0.43	0.36
Days on feed	162.0	153.5	152.0	152.0	6.39	0.39	0.52	0.52

^1^Adjusted with a common average dressing percentage (62.71%) from the trial.

^2^Effect of SBH inclusion.

^3^Effect of CaO addition.

^4^Fiber × Alkali.

^a,b^Values within a row without common superscripts differ (*P* ≤ 0.05).

Carcass characteristics are presented in Table 3. Hot carcass weight and fat thickness were not impacted by SBH inclusion (*P* ≥ 0.79), but yield grade tended (*P* = 0.08) to be greatest in steers fed SBH rations. Addition of CaO did not affect hot carcass weight, fat thickness, or yield grade (*P* ≥ 0.37). However, an interaction was noted where addition of CaO increased hot carcass weight (*P* = 0.04), yield grade (*P* = 0.03) and tended to increase fat thickness (*P* = 0.08), in steers fed diets with no SBH, but decreased these traits in steers fed diets that contained SBH. Dressing percentage, LM area, KPH%, and marbling score did not differ because of SBH inclusion or CaO addition and an interaction did not occur (*P* ≥ 0.14). However, steers fed SBH tended to produce carcasses with a greater percentage of animals grading Select (*P* = 0.09), and also resulted in a lower number of carcasses grading average Choice (*P* = 0.02).

**Table 3.** Effect of SBH inclusion or CaO addition on carcass characteristics of steers

	Treatments							
	No SBH		SBH			*P*-value		
	No CaO	CaO	No CaO	CaO	SE	Fiber^1^	Alkali^2^	F × A^3^
Hot carcass weight, kg	381^a^	394^b^	390^ab^	384^ab^	4.1	0.98	0.48	0.04
Dressing, %	62.3	63.4	62.7	62.6	0.55	0.72	0.37	0.30
Fat thickness, cm	1.12^a^	1.35^b^	1.27^ab^	1.14^ab^	0.094	0.79	0.56	0.08
L. dorsi area, cm^2^	87.1	86.5	86.5	87.1	0.84	0.95	0.66	0.43
Kidney, pelvic, heart fat, %	1.9	1.95	1.88	1.91	0.046	0.60	0.46	0.83
Yield grade	2.86^a^	3.26^b^	3.12^ab^	2.93^a^	0.115	0.08	0.37	0.03
Marbling score	360.7	412.5	371.4	345	24.3	0.27	0.61	0.14
Quality grade distribution								
Select, %	19.0	7.0	24.0	25.0	6.0	0.09	0.44	0.32
Choice^−^, %	53.0	43.0	53.0	61.0	10.0	0.40	0.90	0.42
Choice^0^, %	22.0	32.0	8.0	7.0	7.0	0.02	0.51	0.46
Choice^+^, %	7.0	7.0	7.0	4.0	5.0	0.71	0.71	0.75
Prime, %	0.0	11.0	9.0	4.0	5.0	0.90	0.55	0.12

^1^Effect of SBH inclusion.

^2^Effect of CaO addition.

^3^Fiber × Alkali.

^a,b^Values within a row without common superscripts differ (*P* ≤ 0.05).

### Experiment 2

**Figure 1.**
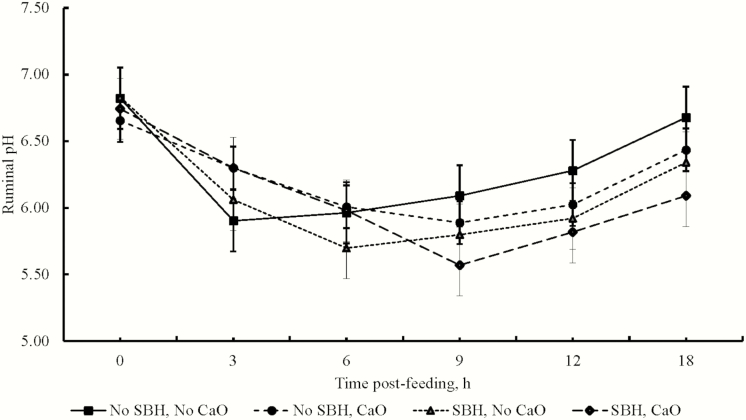
Effect of SBH inclusion and CaO addition on ruminal pH over time. Steers were fed diets with: 1) no SBH and 0% CaO, 2) no SBH and 1% CaO, 3) 30% SBH and 0% CaO, and 4) 30% SBH with 1% CaO. Ruminal pH did not differ because of SBH inclusion or CaO addition (*P* ≥ 0.15). Steers fed SBH tended to have lower ruminal pH from 9 to 18 h postfeeding (fiber × time; *P* = 0.10). Calcium oxide delayed the nadir of ruminal pH from 6 h postfeeding without CaO addition to 9 h postfeeding with CaO addition (alkali × time, *P* = 0.007).

Ruminal pH data are presented in Fig 1. Inclusion of SBH tended to lower ruminal pH for a longer time after feeding (fiber × time interaction, *P* = 0.10). Addition of CaO delayed ruminal pH nadir (alkali × time interaction, *P* = 0.007). No differences in ruminal pH of steers were detected at any individual time point for either SBH inclusion (*P* ≥ 0.15) or CaO addition (*P* ≥ 0.18), and SBH inclusion and CaO addition did not interact (*P* ≥ 0.36).

Ruminal fluid VFA data are presented in Table 4. There were no treatment × time interactions for any VFA, thus only treatment means are presented in Table 4. Total ruminal VFA concentrations (mM) were greater for steers fed SBH rations and greater for steers fed diets with CaO added (*P* < 0.01). Ruminal acetate concentrations were greater with SBH inclusion (*P* < 0.01) and tended to be greater with CaO addition (*P* = 0.09). Ruminal acetate:propionate was greater for steers fed diets with SBH (*P* < 0.01). Ruminal concentrations of formate, propionate, butyrate, isobutyrate, valerate, and isovalerate did not differ with SBH inclusion or CaO addition (*P* ≥ 0.16). No interactions were detected between SBH inclusion and CaO addition for any VFA measurements (*P* ≥ 0.16), with the exception of valerate. Ruminal valerate concentrations tended to decrease with CaO addition in SBH free diets, and increase with CaO addition in SBH diets (*P* = 0.09).

**Table 4.** Effect of SBH or CaO on ruminal VFAs

	Treatments							
	No SBH		SBH			*P*-value		
	No CaO	CaO	No CaO	CaO	SE	Fiber^1^	Alkali^2^	F × A^3^
Total VFA, mM	102.2	118.2	125.2	152.3	7.41	<0.001	<0.01	0.46
Formate, mM	1.7	2.0	1.9	1.8	0.16	0.93	0.37	0.18
Acetate, mM	56.2	66.0	78.9	100.5	8.22	<0.01	0.09	0.49
Propionate, mM	29.6	29.7	28.6	33.1	4.60	0.79	0.63	0.65
Acetate:propionate	2.1	2.5	3.0	3.1	0.20	<0.01	0.19	0.52
Butyrate, mM	8.1	13.5	10.1	10.1	1.77	0.69	0.16	0.16
Isobutyrate, mM	0.95	1.16	0.82	0.80	0.165	0.17	0.57	0.52
Valerate, mM	1.47	1.12	0.86	1.23	0.209	0.24	0.96	0.09
Isovalerate, mM	2.2	2.4	2.0	2.1	0.35	0.51	0.73	0.93

^1^Effect of SBH inclusion.

^2^Effect of CaO addition.

^3^Soybean hull inclusion × CaO addition.

Diet digestibility data are presented in Table 5. Inclusion of SBH did not affect DM, OM, or CP intake, but did increase NDF and ADF intake (*P* < 0.0001). Addition of CaO did not influence any intake parameter and there were no interactions that occurred for DM, OM, CP, NDF, or ADF intake (*P* ≥ 0.40). Inclusion of SBH did not influence DM, OM, or CP apparent digestibility, but apparent digestibility of NDF and ADF increased for steers fed SBH diets (*P* < 0.001 and *P* < 0.0001 for NDF and ADF, respectively). Calcium oxide addition to diets increased apparent digestibility of DM (*P* = 0.04), OM (*P* = 0.05), NDF (*P* = 0.02), and ADF (*P* = 0.02), but not CP (*P* ≥ 0.29). Inclusion of SBH and addition of CaO did not interact to influence apparent digestibility of any nutrient (*P* ≥ 0.18).

**Table 5.** Effect of SBH inclusion or CaO addition on diet digestibility

	Treatments							
	No SBH		SBH			*P*-value		
	No CaO	CaO	No CaO	CaO	SE	Fiber^1^	Alkali^2^	F × A^3^
Intake, kg/d								
DM	8.7	9.0	8.7	9.2	0.49	0.92	0.46	0.85
OM	8.2	8.5	8.1	8.6	0.46	0.93	0.40	0.85
CP	228.9	237.4	230.5	244.3	12.93	0.75	0.41	0.84
NDF	2.8	2.9	4.3	4.6	0.21	<0.0001	0.43	0.74
ADF	1.2	1.2	2.3	2.4	0.11	<0.0001	0.46	0.68
Apparent digestibility, %								
DM	78.3	81.8	79.4	82.5	1.34	0.52	0.04	0.88
OM	80.0	83.5	81.2	83.8	1.32	0.58	0.05	0.75
CP	80.5	82.3	81.5	82.8	1.37	0.58	0.29	0.88
NDF	62.9	71.7	76.7	79.8	2.03	<0.001	0.02	0.19
ADF	55.3	64.3	74.9	77.9	2.08	<0.0001	0.02	0.18

^1^Effect of SBH inclusion.

^2^Effect of CaO addition.

^3^Fiber × Alkali.

Digestion kinetics are presented in Table 6. Inclusion of SBH did not affect ruminal rate (*k*_d_) or extent of DM digestion (*P* ≥ 0.28). In addition, fractional outflow of particulate matter from the reticulorumen (*k*_1_) and from the cecum-colon (*k*_2_), liquid rate of passage (*k*_L_), and mean retention time were not affected by SBH inclusion (*P* ≥ 0.26). Addition of CaO to diets increased *k*_d_ (*P* = 0.05); however, an interaction occurred with SBH inclusion for *k*_d_ (*P* = 0.01), where addition of CaO increased *k*_d_ when SBH were included in the diet, but decreased *k*_d_ when SBH were not included in the diet. Extent of digestion, *k*_1_, *k*_2_, and *k*_L_ were not affected by CaO addition to diets and no interaction was detected (*P* ≥ 0.15). Addition of CaO tended to increase mean retention time (*P* = 0.09).

**Table 6.** Effect SBH inclusion or CaO addition on kinetics of digestion

	Treatments							
	No SBH		SBH			*P*-value		
	No CaO	CaO	No CaO	CaO	SE	Fiber^1^	Alkali^2^	F × A^3^
Degradation (*n* = 2 animals)								
*k*_d_, proportion per h^4^	3.07	2.84	2.70	3.14	0.300	0.94	0.05	0.01
Extent, %	68.7	73.8	73.6	80.7	4.85	0.28	0.19	0.40
Passage (*n* = 4 animals)								
*k*_1_, proportion per h^5^	0.027	0.019	0.024	0.023	0.0031	0.83	0.15	0.32
*k*_2_, proportion per h^6^	0.076	0.088	0.083	0.089	0.0089	0.70	0.34	0.72
*k*_L_, proportion per h^7^	0.058	0.065	0.058	0.056	0.0039	0.26	0.54	0.28
Mean retention time, h	57.6	78.7	62.6	71.2	7.38	0.87	0.09	0.43

^1^Effect of SBH inclusion.

^2^Effect of CaO addition.

^3^Fiber × Alkali.

^4^ *k*_d_ is the rate of degradation.

^5^ *k*_1_ is the fractional outflow rate from the reticulorumen (the smaller rate constant for the pool with a longer retention time).

^6^ *k*_2_ is the fractional outflow rate from the hindgut (the larger rate constant for the pool with a shorter retention time).

^7^ *k*_L_ is the liquid passage rate.

Data on nitrogen balance are shown in Table 7. Nitrogen intake of steers did not differ because of SBH inclusion or CaO addition. However, total nitrogen excretion tended to decrease with SBH inclusion (*P* = 0.07) and CaO addition (*P* = 0.07). Fecal DM and nitrogen excretion, and percentage of total nitrogen excreted in feces did not differ because of SBH inclusion or CaO addition (*P* ≥ 0.19). Urinary volume tended to decrease with CaO addition (*P* = 0.10), and was not affected by SBH inclusion (*P* = 0.44). Urinary excretion of total N (g/d) was lower for steers fed SBH diets (*P* = 0.05) and decreased with CaO addition (*P* = 0.05). Total nitrogen excreted in urine as a percent of total nitrogen excreted by steers did not differ with SBH inclusion or CaO addition (*P* ≥ 0.26). Urinary urea nitrogen was lower for steers fed SBH diets (*P* = 0.02), and was not affected by CaO addition (*P* = 0.30). Urinary ammonia nitrogen tended (*P* = 0.09) to increase and urine pH increased (*P* = 0.01) with SBH inclusion and neither were impacted by addition of CaO (*P* ≥ 0.22). No interactions between SBH inclusion and CaO addition were detected for any of the nitrogen balance parameters (*P* ≥ 0.59).

**Table 7.** Effect of SBH inclusion or CaO addition on nitrogen balance

	Treatments							
	No SBH		SBH			*P*-value		
	No CaO	CaO	No CaO	CaO	SE	Fiber^1^	Alkali^2^	F × A^3^
N intake, g/d	228.9	237.4	230.5	244.3	12.93	0.75	0.41	0.84
N excretion, g/d	141.7	127.9	127.7	111.7	7.21	0.07	0.07	0.89
Fecal excretion								
DM, kg/d	1.9	1.6	1.8	1.6	0.16	0.61	0.19	0.85
Total N, g/d	44.6	42.1	42.5	41.4	3.90	0.72	0.66	0.86
Total N, % N excretion	31.5	32.6	33.3	37.8	2.93	0.26	0.36	0.59
Urinary excretion								
Volume, L/d	9.4	7.3	8.2	6.9	0.94	0.44	0.10	0.67
Total N, g/d	97.1	85.7	85.2	70.3	5.93	0.05	0.05	0.77
Total N, % N excretion	68.5	67.4	66.7	62.2	2.93	0.26	0.36	0.59
Urea N, g/d	74.3	68.7	53.8	39.5	8.98	0.02	0.30	0.64
Ammonia N, g/d	22.7	15.7	31.8	30.8	6.33	0.09	0.55	0.64
Urine pH	7.1	7.7	8.2	8.4	0.30	0.01	0.22	0.68

^1^Effect of SBH inclusion.

^2^Effect of CaO addition.

^3^Fiber × Alkali.

## DISCUSSION

The effect that SBH inclusion had on DMI and ADG in the present study are in agreement with Ferreira et al. (2011) who reported that replacing up to 45% of the corn in feedlot lamb diets with SBH increased DMI and had no effect on ADG. Our results are also in agreement with Mueller et al. (2011) who demonstrated that replacing corn with SBH in an oat silage-based diet increased DMI and had no effect on ADG of newly received feedlot calves. Ludden et al. (1995) observed that increasing SBH inclusion in starch-based feedlot diets increased DMI and decreased ADG. However, Bittner et al. (2016) noted that SBH fed in combination with modified dry DGS or wet DGS produced similar or greater gains than corn-based diets. Addition of SBH increased DMI and did not depress ADG in the present study likely because of positive associative effects with the other dietary ingredients and the fiber was more thoroughly digested, as evidenced by the increases in apparent digestibility of NDF and ADF with SBH inclusion. Soybean hulls are 74% NDF (NRC, 1996), however, the energy value of SBH in rations can vary depending on other dietary components. Ludden et al. (1995) demonstrated the SBH fed in a starch-based feedlot ration resulted in SBH providing only 74% the energy of corn. Others have observed that SBH fed in high-fiber diets can provide energy comparable to that of corn (Anderson et al., 1988; Garcés-Yépez et al., 1997). Bittner et al. (2016) demonstrated that when SBH were fed in combination with DGS, SBH can provide from 99% to 106% of the energy content of corn in feedlot diets as a result of potential positive associative effects in the rumen. Further, addition of SBH to rations containing low-quality forages has been shown to result in greater digestibility (Highfall et al., 1987; Galloway et al., 1993; Fieser and Vanzant, 2004; Orr et al., 2008). Specifically, Orr et al. (2008) concluded that when SBH replaced a portion of corn in diets of steers fed bermudagrass hay apparent NDF and ADF digestibilities increased. It is likely in the present study that SBH provided more energy than the corn it replaced because of a positive associative effect with the dried DGS in the diet.

Calcium oxide addition increased ADG in the first half of the present study for steers fed SBH free diets. Further, gain:feed in the second period was increased by CaO in cattle fed SBH free diets. Morrow et al. (2013) observed that treating dried DGS with 2% NaOH in diets of feedlot lambs tended to increase ADG and DMI, as a result of the neutralization of the inherent acidity of dried DGS. Similarly, Nuñez et al. (2014) reported a linear increase in DMI and gain:feed with increasing CaO inclusion up to 2.4%. However, a quadratic response was present where ADG increased from 0% to 0.8% CaO inclusion but decreased from 1.6% to 2.4% inclusion (Nuñez et al., 2014). Schroeder et al. (2014a) demonstrated that treating dried DGS or modified dry DGS with 1.2% CaO decreased DMI, with no effect on ADG, resulting in an increased gain:feed. Schroeder et al. (2014a) attributed the decrease in DMI with CaO inclusion to a decrease in meal sizes from 0 to 3 h postfeeding without an increase in meal frequency. The improvement in first half ADG from CaO in the current study is most likely due to increased ruminal pH and improved apparent NDF and ADF digestibilities. Addition of hydrolytic chemical agents, such as CaO, to highly lignified feedstuffs have been shown to break bonds between lignin and structural carbohydrates, resulting in increased microbial fermentation of the feedstuff (Jung and Deetz, 1993). Although the roughages in the present study were not pretreated with a strong base, increases in apparent digestibilities of DM, OM, NDF, and ADF from direct addition of CaO to rations in the current study aligns with previous research (Nuñez et al., 2014; Lancaster et al., 2019). Improved fiber digestibility from direct addition of alkali to rations may occur through degradation of the linkages between lignin and hemicellulose in addition to increased ruminal pH (Nuñez et al., 2014). Stroud et al. (1985) demonstrated that addition of 1% sodium bicarbonate to a cracked corn-based diet improved total tract DM and NDF digestion. Additionally, adding sodium bicarbonate to steam-flaked corn and sorghum diets increased total tract ADF digestion (Zinn, 1991). Felix et al. (2012a) reported that addition of 2% sodium hydroxide to 60% DDGS diets increased NDF degradation, while Nuñez et al. (2014) observed that CaO addition increased NDF and ADF apparent digestibilities with increasing amounts of CaO addition. Addition of Ca(OH)_2_ has also enhanced fiber digestibility in feedlot steers that were fed diets containing 60% DDGS and stover (Lancaster et al., 2019). Despite what appears to be a greater improvement in fiber digestibility in diets with no SBH as a result of CaO addition, there was no interaction in the current study.

Calcium oxide addition to SBH diets actually decreased overall ADG in the current study, even though there were trends for increased fiber digestibility and ruminal VFA concentrations. The interactions that occurred for hot carcass weight, fat thickness, and yield grade, where CaO increased these traits in cattle fed no SBH but decreased them in cattle fed SBH is linked to the interaction that occurred for overall carcass-adjusted ADG. It is unclear why carcass-adjusted ADG was depressed in cattle fed SBH and CaO. The SBH + CaO treatment in the present study had the greatest concentration of total VFA which may be indicative of decreased VFA absorption. Volatile fatty acid absorption is complex and influenced by many factors (rate of fermentation, meal size and frequency, rumen fill, and absorption pathway). It is possible that absorption of VFA across the rumen wall may have been inhibited by the combination of SBH and CaO. On the other hand, the increase in total ruminal VFA and acetate concentration from SBH inclusion and CaO addition in the present study may be due to an increase in ruminal fiber fermentation as a result of alterations in the pH profile of the rumen. Distillers grains with solubles can lower ruminal pH below 5 (Felix and Loerch, 2011) because of sulfuric acid addition during the ethanol fermentation process (McAloon et al., 2000). Lower ruminal pH inhibits the activity and growth of cellulolytic bacteria (Mould and Orskov, 1983), leading to decreased fiber fermentation. In the present study, cattle fed the SBH diets tended to have a lower ruminal pH from 6 to 18 h postfeeding, and cattle fed CaO had an increased ruminal pH from 3 to 6 h postfeeding. Martin and Hibberd (1990) reported that increasing SBH inclusion in a cottonseed meal diet for beef heifers resulted in a decreased ruminal pH, which was attributed to greater VFA production. Similarly, Weidner and Grant (1994) observed that replacing a portion of alfalfa and corn silage with SBH decreased ruminal pH and acetate:propionate in lactating dairy cows. In contrast, Orr et al. (2008) reported no effect on ruminal pH when SBH were supplemented to steers receiving bermudagrass hay. Further, steers fed stover with added SBH were shown to have increased ruminal pH compared with steers supplemented with corn (Anderson et al., 1988). Changes in ruminal pH in the present study as a result of CaO addition are consistent with previous studies. Nuñez et al. (2014) demonstrated that the addition of CaO at 1.6% or 2.4% DM to a 60% DDGS ration delayed the postfeeding decline in pH. Addition of Ca(OH)_2_ increased ruminal pH and delayed pH nadir in feedlot steers fed 60% DDGS diets that included stover (Lancaster et al., 2019). It is possible that changes in pH and VFA production in cattle fed alkali may change feeding pattern. Schroeder et al. (2014b) reported an increase in ruminal pH at 3 h postfeeding with 1.2% CaO addition to a 48.8% dried DGS ration and observed that meal sizes decreased from 0 to 3 h postfeeding, with no increase in meal frequency (Schroeder et al., 2014a).

Our data suggest that CaO increased *k*_d_ in SBH diets, but decreased *k*_d_ in SBH free diets. Lancaster et al. (2019) observed that Ca(OH)_2_ increased *k*_d_ in 60% DDGS diets that contained corn silage, stover, or additional corn. Previous work suggests that high-fiber energy feeds like SBH to diets can decrease rates of degradation. For example, Moore et al. (1990) demonstrated that replacing half the alfalfa hay in a 65% concentrate, 35% alfalfa hay diet with cottonseed hulls resulted in decreased *k*_d_, increased *k*_p_, and no change in extent of digestion. Hintz et al. (1964) reported an increase in ruminal rate of passage in lambs when SBH were fed alone, as compared with when SBH was fed with mixed hay. Digestibility is inherently complex in that it is dependent on several factors, including the amount and composition of fiber in the diet, intake level, passage rate, and particle size (Van Soest, 1982). Forage substrates differ in rates of hydration or rates of chemical or physical alteration before enzymatic degradation (Mertens and Ely, 1982). It is possible that addition of CaO to the SBH ration removed some chemical or physical inhibitors in the SBH or increased the capability of the fibers to swell via hydration which is needed before enzymes can contact and react with fiber molecules (Allen and Mertens, 1988). Although CaO addition to the ration did not impact solid or liquid passage rates, mean retention time tended to be increased by CaO addition in the present study, indicating that rates of passage may have been slower. Aines (1985) observed that pretreating wheat straw with 4% or 8% Ca(OH)_2_ and feeding 90% wheat straw diets did not have an effect on solid or liquid passage rates. However, Lancaster et al. (2019) noted that even though Ca(OH)_2_ did not affect solid passage rates or mean retention time, Ca(OH)_2_ did increase liquid passage rates in 60% DDGS diets that contained corn silage, stover, or additional corn. Similarly, Oliveros et al. (1993) also reported that solid passage rate was not impacted while liquid passage rate was increased when corn stover in 90% corn stover diets was pretreated with 40 g/kg of Ca(OH)_2_. In addition, Casperson (2017) observed that replacement of a portion of haylage and corn silage with 9.6% of the diet DM as corn stover pretreated with 6.2% Ca(OH)_2_ did not affect solid passage rate but increased liquid passage rate in lactating dairy cows. Increased liquid passage rate as a result of alkaline treatment would increase the amount of soluble nutrients carried postruminally and shift the site of starch digestion to the small intestine, which has been shown to improve feed efficiency (Harmon and McLeod, 2001).

In the present study, urinary N excretion was lower for steers consuming SBH diets, indicating that N utilization was increased with SBH inclusion. Decreased urinary N excretion because of SBH inclusion indicates that there may have been more ruminal protein fermentation with SBH inclusion, even though total tract apparent digestibility of CP was not impacted by SBH inclusion. In agreement, Orr et al. (2008) observed that SBH inclusion in the diets of steers fed bermudagrass increased ruminal ammonia N and improved ruminal N utilization. In the present study, urine pH was increased with SBH inclusion, despite a lower ruminal pH for cattle fed SBH. It appears that replacing corn with SBH decreases dietary anion concentrations and/or increases cations. Felix et al. (2012b) reported a linear decrease in urine pH with increasing dietary acid load from greater DDGS inclusion in diets of feedlot lambs. Calcium oxide and Ca(OH)_2_ have been observed to increase urine pH in 60% DDGS rations (Nuñez et al, 2014; Lancaster et al., 2019), suggesting that added dietary alkali improved the acid–base balance of steers in these studies. Although CaO seems to increase urine pH in the present study, it did not differ from urine pH in cattle not fed CaO possibly because only 30% DDGS were fed, with a lower dietary acid load, resulting in less need for regulation of acid–base balance. It may be possible that blood pH was too alkaline in steers fed CaO and SBH, explaining the decreased gain that was seen in these cattle.

In conclusion, when SBH replaces a portion of corn in cattle fed dry DGS, positive associative effects increase ruminal fiber digestibility and increases gain. Addition of CaO to corn, dry DGS, and corn stover rations, without added SBH improved digestibility and animal performance. However, although ruminal parameters were improved, the addition of CaO did not improve growth of feedlot cattle fed a combination of SBH, dry DGS, corn, and stover.
